# Role of viral coinfections in asthma development

**DOI:** 10.1371/journal.pone.0189083

**Published:** 2017-12-05

**Authors:** Maria Luz Garcia-Garcia, Cristina Calvo, Sara Ruiz, Francisco Pozo, Victoria del Pozo, Laura Remedios, Nadia Exposito, Ana Tellez, Inmaculada Casas

**Affiliations:** 1 Pediatrics Department, Severo Ochoa Hospital, Leganés, Alfonso X El Sabio University, Madrid, Spain; 2 Traslational Research Network in Pediatric Infectious Diseases (RITIP); 3 TEDDY Network (European Network of Excellence for Pediatric Clinical Research); 4 Pediatrics Department, La Paz Hospital, Alfonso X El Sabio University, Madrid, Spain; 5 Respiratory Virus and Influenza Unit, National Microbiology Center (ISCIII), Madrid, Spain; 6 Department of Immunology, CIBER de Enfermedades Respiratorias (CIBERES), IIS-Fundación Jiménez Díaz, Madrid, Spain; Kliniken der Stadt Köln gGmbH, GERMANY

## Abstract

**Background:**

Viral respiratory infections, especially acute bronchiolitis, play a key role in the development of asthma in childhood. However, most studies have focused on respiratory syncytial virus or rhinovirus infections and none of them have compared the long-term evolution of single versus double or multiple viral infections.

**Objective:**

Our aim was to compare the frequency of asthma development at 6–8 years in children with previous admission for bronchiolitis associated with single *versus* double or multiple viral infection.

**Patients & methods:**

A cross-sectional study was performed in 244 children currently aged 6–8 years, previously admitted due to bronchiolitis between September 2008 and December 2011. A structured clinical interview and the ISAAC questionnaire for asthma symptoms for 6-7-year-old children, were answered by parents by telephone. Specimens of nasopharyngeal aspirate for virological study (polymerase chain reaction) and clinical data were prospectively taken during admission for bronchiolitis.

**Results:**

Median current age at follow-up was 7.3 years (IQR: 6.7–8.1). The rate of recurrent wheezing was 82.7% in the coinfection group and 69.7% in the single-infection group, p = 0.06. The number of wheezing-related admissions was twice as high in coinfections than in single infections, p = 0.004. Regarding the ISAAC questionnaire, 30.8% of coinfections versus 15% of single infections, p = 0.01, presented “wheezing in the last 12 months”, data that strongly correlate with current prevalence of asthma. “Dry cough at night” was also reported more frequently in coinfections than in single infections, p = 0.02. The strongest independent risk factors for asthma at 6–8 years of age were: age > 9 months at admission for bronchiolitis (OR: 3.484; CI95%: 1.459–8.317, p:0.005), allergic rhinitis (OR: 5.910; 95%CI: 2.622–13.318, p<0.001), and viral coinfection-bronchiolitis (OR: 3.374; CI95%: 1.542–7.386, p:0.01).

**Conclusions:**

Asthma at 6–8 years is more frequent and severe in those children previously hospitalized with viral coinfection-bronchiolitis compared with those with single infection. Allergic rhinitis and older age at admission seem also to be strong independent risk factors for asthma development in children previously hospitalised because of bronchiolitis.

## Introduction

There is strong evidence that respiratory viruses play a key role in the development of asthma in children. Classic studies by Sigurs et al. showed that respiratory syncytial virus (RSV) bronchiolitis, severe enough to require hospitalization, is a risk factor for asthma at the age of 7, 13 and 18 [1,2,3]. ng asthma at 6 years being more than four-fold higher compared with HRV- negative cases [4,5]. Other studies have reported that early human metapneumovirus (hMPV) infection during infancy is also an independent risk factor for the development of preschool asthma [6]]. However, most studies have focused on RSV or HRV infections and only one of thm has evaluated the long-term evolution of viral coinfections, although focused exclusively on human bocavirus (HBoV) and HRV coinfections in 3 to 35 month-old children with their first or second wheezing episode [7].

Our main objective was to compare the frequency of asthma and other respiratory symptoms, at 6–8 years of age, in children with previous admission with bronchiolitis associated with viral co-infection *versus* simple viral infection.

## Patients and methods

This is a substudy of an ongoing prospective investigation of respiratory tract infections in children, approved by The Medical Ethics Committee.

### Clinical assessment

Inclusion criteria were children 6–8 years of current age, with a previous hospitalization with bronchiolitis at 0–24 months of age, between September 2008 and December 2011.

During the hospital stay a physician filled out a study-questionnaire with the following variables: age, sex, month of admission, clinical diagnosis, history of prematurity, need for oxygen therapy–evaluated via transcutaneous oxygen saturation -, axillary temperature (≥ 38°C), presence of infiltrates and/or atelectasis in chest X-rays, administration of antibiotic therapy, length of hospital stay, total white blood cell (WBC) count, C-reactive protein (CRP) serum levels and blood culture results (for those cases where such tests were performed) and results of the virological study.

Parents were contacted by phone between October 2016 and January 2017, and were invited to take part in the study. A telephone interview based on a structured questionnaire was performed, to obtain information on wheezing episodes, related hospital admissions, physician-diagnosed atopic dermatitis, allergic rhinitis, food allergy, use of bronchodilators and maintenance medication for asthma. Information on pet contacts, parental smoking habits, presence of allergy, eczema and asthma in first order family members (mother, father or siblings) that had been diagnosed by a medical doctor was also recorded.

Furthermore, the present investigation used the ISAAC questionnaire for asthma symptoms for 6-7-year-old children, previously validated and translated to Spanish [8], which was answered by parents over the phone.

The researchers were blinded to the status of the child when the interviews were performed. Informed consent was obtained from parents or legal guardians. Exclusion criteria were refusal to participate.

### Virus detection

Specimens consisted of nasopharyngeal aspirates (NPA) that were taken from each patient at admission. Each specimen was sent for virological investigation to the Respiratory Virus and Influenza Unit at the National Microbiology Center (ISCIII, Madrid, Spain). NPAs were processed within 24 hours after collection. Upon receipt, three aliquots were prepared and stored at -70°C. Both the reception and the NPA sample processing areas were separate from those defined as working areas.

#### Polymerase chain reactions (PCR) methods for detection of sixteen respiratory viruses

Three reverse transcription (RT)-nested PCR assays were performed to detect a total of sixteen respiratory viruses. In these assays, the reverse transcription and first amplification round were carried out in a single tube using the Qiagen OneStep RT-PCR kit (Qiagen). Influenza A, B and C viruses were detected by using primer sets only to amplify influenza viruses in a multiplex PCR assay as previously described [9]. A second multiplex PCR was used to detect parainfluenza viruses 1 to 4 (PIV), human coronaviruses (CoV) 229E and OC43, enteroviruses (EV) and HRV [10]. Presence of RSV A and B types, hMPV, HBoV and adenoviruses (AdV) were investigated by a third multiplex RT-nested PCR-BRQ method [11] (S1 Table).

### Clinical definitions

#### Bronchiolitis

All the classic criteria, present in an initial episode of acute onset expiratory dyspnea with previous signs of viral respiratory infection—whether or not this was associated to respiratory distress or pneumonia—were applied in diagnosing bronchiolitis [12].

The term *recurrent wheezing* was used to imply more than one episode of wheezing verified by a physician.

The children who answered affirmatively to question 2 of the ISAAC questionnaire, "wheezing in the last 12 months", being the one that in the validation studies has shown a better correlation with the current prevalence of asthma, were considered to have *asthma* [13].

#### Allergic rhinitis

Was defined as rhinitis appearing at least twice after exposure to a particular allergen and unrelated to infection.

#### Viral coinfection

Was defined as the detection of more than one viral pathogen in the same sample.

### Statistical analysis

Continuous variables were described using mean and standard deviation. Categorical variables were described using absolute and relative frequencies. Clinical characteristics of patients with simple infections were compared with those associated with coinfections.

To compare qualitative variables, Chi^2^ test or Fisher's exact test was used if there were ≤5 items of data in a cell. For quantitative variables, as all of them followed a normal distribution, the means were compared using the Students´s T to compare two groups. A two-sided value of *P* < 0.05 was considered statistically significant.

Multivariate stepwise logistic regression analysis was used to calculate the adjusted odds ratios (OR) with 95% confidence intervals for estimating the association between different factors and asthma. All analyses were performed using the Statistical Package for the Social Sciences (SPSS), Version 21.0.

## Results

Of the 351 children previously admitted with bronchiolitis, with positive viral detection and current age between 6 and 8 years, 244 (52 coinfections and 192 single infections) could be located and agreed to participate in the study.

Clinical characteristics during admission for bronchiolitis are presented in Table 1. No significant clinical differences could be detected between both groups, coinfections and single infections, in terms of age at admission, gender, prematurity, fever, hypoxia or length of hospital stay. The most frequently identified viruses in single infections were: RSV (100, 52%), HRV (29, 15%), hMPV (15, 8%), AdV (5, 2.6%) and HBoV (3, 1.6%). The most frequent coinfections were RSV + HRV (15, 29%) and RSV + HBoV (12, 23%).

**Table 1:** Clinical characteristics at admission of infants with bronchiolitis associated with viral coinfection *versus* single infection.

Clinical feature	Viral Coinfection N = 52	Single infection N = 192	P-value
Age* **(months)**	5.4 (4.1)	5.3 (5.7)	0.942
**Male**	29 (55.8%)	87 (45.3%)	0.180
**Prematurity**	6 (11.5%)	24 (12.6%)	0.842
**Temperature > 37.9°C**	30 (57.7%)	91 (47.4%)	0.188
**Hypoxia (SatO2<95%)**	26 (50%)	121 (63.4%)	0.080
**PICU admission**	1(1.9%)	8(4.2%)	0.446
**Abnormal X-ray**	13 (34%)	55 (38%)	0.652
**Antibiotic treatment**	10 (19%)	26 (13.5%)	0.305
Highest temperature*	38.6 (0.6)	38.7 (0.5)	0.428
Leucocytes*;cells/mm3	13,638 (5,656)	14,038 (13,840)	0.892
C reactive protein*; mg/L	29 (22.3)	25.8 (31.1)	0.062
Days of hospital stay*	4.2 (2.8)	4.5(2.8)	0.457
Days of fever*	3.1 (2.0)	2.6 (1.8)	0.539
Days of hypoxia*	3.7 (2.6)	3.3 (2.7)	0.507

*Mean (Standard deviation)

PICU: paediatric intensive care unit.

With respect to comparability between both groups, no differences were found between coinfections and single infections regarding allergic rhinitis, atopic dermatitis or food allergy (Table 2). Several possible hereditary and environmental factors for the development of recurrent wheezing or asthma were also evaluated. No differences were observed in the family history of asthma or atopy. Nearly one-third of children were exposed to tobacco smoke, with similar percentages in both groups (Table 2).

**Table 2:** Evaluation of background factors in 52 children with viral coinfection bronchiolitis and 192 simple infection bronchiolitis.

BACKGROUND FACTOR	VIRAL COINFECTION N = 52	SINGLE INFECTION N = 192	P-value
Current age* **(years)**	7.4 (0.9)	7.4 (0.9)	0.799
**Breast feeding**	43(82.7%)	154(80%)	0.687
**Atopic dermatitis**	22(42.3%)	74(38.5%)	0.622
**Food allergy**	5(9.6%)	19(9.9%)	0.952
**Allergic rhinitis**	7(13.5%)	38(19.8%)	0.296
**Asthma**			
**Mother**	2(3.8%)	22(11.5%)	0.102
**Father**	4(7.7%)	23(13.5%)	0.255
**Siblings**	7(13%.5)	18(9.4%)	0.389
**Atopy**			
**Mother**	6(11.5%)	32(16.7%)	0.366
**Father**	6(11.5%)	27(14%)	0.637
**Siblings**	9(17.3%)	34(17.7%)	0.946
**Smoking**			
**Mother**	16(30.8%)	46(24%)	0.317
**Father**	14(27%)	65(34%)	0.343

*Mean (Standard deviation)

Median current age at follow-up contact was 7.3 years (IQR: 6.7–8.1), with a slight predominance of females (52.5%). The rate of recurrent wheezing was 82.7% in the coinfection group and 69.7% in the single-infection group, p = 0.06, needing rehospitalization for wheezing 21% and 29% respectively, p = 0.251. The number of wheezing-related admissions was twice as high in coinfections than in single infections, p = 0.004. A similar percentage of children were prescribed inhaled corticosteroids in both groups. However, montelukast was used more frequently in coinfections than in simple infections, p = 0.01 (Table 3).

**Table 3:** Respiratory evolution in children with viral coinfection *versus* single infection bronchiolitis.

	VIRAL COINFECTION N = 52	SINGLE INFECTION N = 192	P-value	OR (CI 95%)
**Recurrent wheezing**	43 (82.7%)	134 (69.7%)	0.060	2.068 (0.946–4.519)
**Wheezing-related admissions**	11 (21%)	56 (29.2%)	0.251	0.652 (0.313–1.358)
**Number of wheezing-related admissions**	4.4 (2.1)	2.2 (1.6)	0.004	
**Asthma treatment**	25 (48%)	68 (35.4%)	0.090	1.688 (0.909–3.136)
**Budesonide**	14 (27%)	46 (24.2%)	0.688	1.153 (0.574–2.315)
**Montelukast**	21 (40.4%)	43 (22.6%)	0.010	2.316 (1.209–4.435)

OR: Odds ratio

CI: Confidence interval

Regarding the ISAAC questionnaire answers, nearly 70% had “ever wheezed”, without significant differences between both groups. However, 30.8% of coinfections versus 15% of single infections, p = 0.01, presented “wheezing in the last 12 months”, data that strongly correlate with current prevalence of asthma. “Dry cough at night” was also reported more frequently in coinfections than in single infections, p = 0.02. The questions: “Wheezing during exercise?” and “Ever had asthma?” were affirmatively answered with similar frequency in both groups. (Table 4).

**Table 4:** Asthma symptoms according ISAAC questionnaire in 52 children with background of viral coinfection bronchiolitis and 192 single infection bronchiolitis.

ASTHMA SYMPTOMS	VIRAL COINFECTION N = 52	SINGLE INFECTION N = 192	P-value	OR (CI 95%)
**Ever Wheezing**	39 (75%)	133 (69.3%)	0.422	1.331 (0.662–2.676)
**Wheezing last 12 months**	16 (30.8%)	29 (15%)	0.010	2.498 (1.229–5.077)
**Limited speech due to wheezing**	5 (9.6%)	4 (4.5%)	0.237	2.234 (0.572–8.725)
**Ever had Asthma**	10 (19.2%)	55 (28.6%)	0.173	0.593 (0.278–1.265)
**Wheezing during exercise**	4 (7.7%)	8 (4.2%)	0.297	1.917 (0.554–6.634)
**Dry Cough at night**	8 (15.4%)	11 (5.7%)	0.021	2.992 (1.136–7.880)

OR: Odds ratio

CI: Confidence interval

In the combined coinfection and single infection group, several possible hereditary and environmental risk factors for asthma at the age of 6–8 were evaluated using a univariate analysis (Table 5). The risk of asthma was increased in children previously hospitalized for bronchiolitis with viral coinfection (OR = 2.498; 95% CI: 1.229–5.077), in those who were older at the time of admission (7.5 ± 8.4 months *versus* 4.8 ± 4.2 months, p = 0.04), and in those with atopic dermatitis (OR = 1.899; 95% CI: 0.980–3.680), allergic rhinitis (OR = 4.139; 95% CI: 1.983–8.639) and food allergy (OR = 3.077; 95%CI:1.222–7.749). Almost 32% of children who were older than 9 months at admission, developed asthma at 6–8 years old, *vs*. 18% of those less than 9 months, p: 0.05. The proportion of asthma in children admitted at > 12 months of age, reached 52.4% *vs*. 17% in those hospitalized with less than 12 months, p< 0.001.

**Table 5:** Univariate tests of possible risk factors for current asthma at age 6–8 years in the compiled coinfection and single infection groups.

RISK FACTORS	P- value	Odds Ratio	CI 95%
**Viral coinfection**	0.010	2.498	1.229–5.077
**Age >12 months at admission**	<0.001	5.274	2.074–13.402
**Age >9 months at admission**	0.050	2.153	0.982–4.719
**Atopic dermatitis**	0.050	1.899	0.980–3.680
**Allergic rhinitis**	<0.001	4.139	1.983–8.639
**Food allergy**	0.013	3.077	1.222–7.749
**Prenatal tobacco exposure**	0.080	1.916	0.917–4.001
**Breast feeding**	0.479	1.377	0.567–3.344
**Prematurity**	0.744	1.177	0.443–3.129
**Day care attendance**	0.862	0.940	0.468–1.889
**Furred pets**	0.656	0.846	0.405–1.767
**Asthma mother**	0.576	1.325	0.493–3.560
**Asthma father**	0.178	1.798	0.760–4.254

OR: Odds ratio

CI: Confidence interval

The risk factors in Table 5 with a p-value < 0.10 were entered in a stepwise logistic regression analysis to estimate which factors were independently related to the development of asthma at 6–8 years. Age > 12 months at admission for bronchiolitis (OR: 7.389; 95%CI: 2.683–20.352, p<0.001), age > 9 months at admission for bronchiolitis (OR: 3.484; CI95%: 1.459–8.317, p:0.005), allergic rhinitis (OR: 5.910; 95%CI: 2.622–13.318, p<0.001), and viral coinfection- bronchiolitis (OR: 3.374; CI95%: 1.542–7.386, p:0.01) were the strongest independent risk factors for asthma at 6–8 years of age.

## Discussion

Our current study shows for the first time to our knowledge, that there is a strong and independent association between severe bronchiolitis associated with viral coinfection and the development of asthma at 6–8 years old.

Viral coinfections are usually detected in up to 30% of children with an acute respiratory tract infection [14]. Our group, in previous studies, found viral coinfections in 22.8% of 318 infants less than 2 years old admitted with bronchiolitis [11] and in 35% of 2,993 children less than 14 years old admitted with lower respiratory tract infection [15]. Our current results confirm that the most frequently identified viral coinfections are dual RSV-HRV and RSV-HBoV infections. Results from cohort studies evaluating the severity of acute respiratory viral coinfections are conflicting. Our group previously found that clinical severity was associated mainly with RSV infections, either alone or in combination with other viruses [15]. However, Asner et al. [16] in a recent systematic review and meta-analysis found no differences in clinical disease severity between viral coinfections and simple infections, although an increased risk of mortality was observed amongst preschool children with coinfections. They conclude that large, prospective, well designed studies with objective outcomes are needed to better understand the clinical significance of viral respiratory coinfections.

Regarding the role of early viral respiratory coinfections in the development of wheezing or asthma, so far, no study evaluating this possible association has been published. Our results confirm that recurrent wheezing and asthma are very common in the first years of life after a severe bronchiolitis. But the likelihood of developing asthma was 2.5 times higher if bronchiolitis was associated with viral coinfection compared to single infections. In addition, the number of rehospitalizations for asthma was also significantly higher in coinfections than in single infections, suggesting that coinfections are associated not only with more frequency of asthma but also with greater severity.

The mechanism by which respiratory infections are associated with subsequent asthma is not fully understood but the different inflammatory responses released after viral infections seem to play a relevant role. The induction of a prevalent Th2-type response, with the production of IL-4, IL-5, and IL-13, results in a less robust and less efficient reaction to the infection, clinically presenting as increased disease severity and risk for future recurrent wheezing [17,18]. Several reports showed that thymic stromal lymphopoietin (TSLP) is a key initiator of allergic airway responses [19] and significant negative correlation has been described between TSLP levels in induced sputum and FEV-1 in asthmatic children [20]. TSLP is secreted by RSV-infected airway epithelial cells (AECs) to promote the activation of dendritic cells (DCs) [21,22]. The activated DCs can then induce a Th2 cell polarization response in the lung, which could contribute the development of asthma in RSV-infected individuals, as well as induction of exacerbations in asthmatic RSV-infected patients. It has been suggested that the ability of RSV to induce TSLP expression by AECs is responsible for the subsequent Th2 aspects of the immune response [23]. Other recent studies performed in young children with HRV infection, have also found higher levels of nasal TSLP [24,25].

On the other hand, various genetic studies in humans have identified both IL-33 and its receptor ST2, as being key regulators in the development of asthma [26] and to have a strong Th2- promoting ability in animal models of asthma [27]. IL-33 is responsible for the immunopathophysiological response observed following neonatal RSV infection in mice. Its presence in nasal aspirates of human infants with severe RSV, together with the finding that by blocking the receptor of interleukin 33 in vivo [28], Th2 responses are greatly reduced, suggest it has a role in disease severity and asthma [29]. Our findings, in a recent study conducted in hospitalized infants with bronchiolitis and in healthy controls, showed that nasal TSLP and IL-33 are detected more frequently in viral coinfections than in single infections. In addition, infants with dual RSV+HRV infection were 9 times more likely to have detectable nasal TSLP and this association was independent of other factors such as age or illness severity [30]. It is worth noting that no patient with bronchiolitis but with negative viral detection had detectable levels of nasal TSLP or IL-33, suggesting that the release of these proteins may be mediated by respiratory viruses.

It can be hypothesized that this stronger TSLP response after viral coinfection bronchiolitis, could stimulate a vigorous production of Th2-associated effector cytokines, such as IL-4, IL-5, IL-13, as was reported in asthmatic adults by Ying et al, that could be associated with higher frequency of wheezing and asthma development later on [31]. As far as we know, only Lukkarinen et al [7] have compared the systemic Th1-type, Th2-type and proinflammatory cytokine profiles in young children with simple versus viral coinfection, being HRV and HBoV1 the viruses involved. They included hospitalized children less than 35 months of age with their first or second wheezing episode. They observed that wheezing children with HRV had higher proinflammatory responses than did those with HBoV1 or those with coinfection HRV+HBoV1, suggesting that HBoV1 may interfere with HRV-induced immune responses. Furthermore, this immunological response was accompanied by the clinical finding that children with HRV+HBoV1 wheeze tended to develop recurrent wheezing later and less often than did those with HRV wheeze. Our previous and current results suggest that an interaction may also occur in RSV and HRV coinfections, but in an opposite way, towards a predominant Th2 response that could increase the development of recurrent wheezing/asthma.

The risk of asthma in our study, like in other cohort studies on hospitalized children with lower respiratory symptoms, was directly dependent on age [4,32]: the adjusted OR was 3.4 for asthma in children with bronchiolitis aged > 9 months and 7.3 for asthma in school age in bronchiolitis children aged > 12 months. However, Lukkarinen et al [33], in a 7-year follow-up found asthma inversely dependent on age, probably because they studied infants with wheezing only, *vs*. bronchiolitis, with or without wheezing, in other studies as in ours. This different inclusion criteria may bias the kind of patients included in the studies.

Rhinitis is a known risk factor for asthma in children [34]. Our results suggest that rhinitis is an independent risk factor for asthma persistence in school-age children with previous severe bronchiolitis. Lauhkonen et al [35] in a prospective follow-up study, in 102 children hospitalised for bronchiolitis, also found that current asthma was associated with prolonged rhinitis and a positive skin prick test at five to seven years. On the other hand, recent reports have demonstrated that the severity of rhinitis and asthma are closely related in children [36]. Thus, as other authors have stated [37], all these results highlight the convenience to assess nasal symptoms in infants and children with recurrent wheezing and asthma.

In conclusion, asthma at the age of 6–8 is more frequent and severe in those children previously hospitalized with viral coinfection bronchiolitis compared with those with single infection. Moreover, viral coinfection, allergic rhinitis and older age at admission seem also to be strong independent risk factors for asthma development in children previously hospitalised because of bronchiolitis. However, given the complexity of the immunological mechanisms involved, more studies are needed to confirm this association and better understand its physiopathological mechanism.

## Supporting information

S1 Table Technical details of multiplex PCR assays used for diagnosis from September 2008 to December 2011. (DOCX)
