# Supplementary material for: Role of viral coinfections in asthma development
Source: PLoS One. 2017 Dec 5;12(12):e0189083. doi: 10.1371/journal.pone.0189083 (PMC5716580; doi:10.1371/journal.pone.0189083)
Supplement: S1 Table — (DOCX) [file pone.0189083.s001.docx]

S1Table. (Supplemental material). Technical details of multiplex PCR assays used for diagnosis from September 2008 to December 2011.

| Amplification step | Name of assay | Name of primer | Sequence (5' to 3') | Gene | Cycling conditions | Amplicon size (bp) |
| --- | --- | --- | --- | --- | --- | --- |
| RT-PCRs |  |  |  |  |  |  |
|  | Multiplex I^1^ Influenza A, B, C | FluAC1 | GAACTCRTYCYWWATSWCAAWGRRGAAAT | NP | 48ºC-45min 95ºC-15min 45 cycles:  94ºC-30sec  50ºC-2min  68ºC-1min | Flu A: 721 FluB: 991 Flu C: 738 |
|  |  | FluB1 | ACAGAGATAAAGAAGAGCGTCTACAA | NP |  |  |
|  |  | FluABC2 | ATKGCGCWYRAYAMWCTYARRTCTTCAWAIGC | NP |  |  |
|  |  |  |  |  |  |  |
|  | Multiplex II^2^ PIV, CoV, EV, RV | 1-PIV13 | AGGWTGYSMRGATATAGGRAARTCATA | HA | 48ºC-45min 94ºC-3min 45 cycles:  94ºC-30sec  55ºC-1.5min  72ºC-1min | PIV1: 635 PIV3: 635 PIV2: 683 PIV4AB: 1070 HCoV 229E: 851 HCoV-OC43 806 EV: 755 RV: 639 |
|  |  | 2-PIV13 | CTWGTATATATRTAGATCTTKTTRCCTAGT | HA |  |  |
|  |  | 1-PIV2 | TAATTCCTCTTAAAATTGACAGTATCGA | HA |  |  |
|  |  | 1-PIV4 | ATCCAGARRGACGTCACATCAACTCAT | 5'NCR-HA |  |  |
|  |  | 2-PIV24 | TRAGRCCMCCATAYAMRGGAAATA | HA |  |  |
|  |  | 1-hCoV | TGTGCCATAGARGAYWTACTTTTT | SP |  |  |
|  |  | 2-hCoV | AACCGCTTKYACCAKCAAYGCACA | SP |  |  |
|  |  | 1-EV/RV | CTCCGGCCCCTGAATRYGGCTAA | 5'NCR-VP4/VP2 |  |  |
|  |  | 2-EV/RV | TCIGGIARYTTCCASYACCAICC | 5'NCR-VP4/VP2 |  |  |
|  |  |  |  |  |  |  |
|  | Multiplex III^3^ RSV, AdV, HMPV^4^,HBoV^5^ | RSVAB1 | ATGGAGYTGCYRATCCWCARRRCAARTGCAAT | F | 48ºC-45min 95ºC-15min 45 cycles:  94ºC-30sec  50ºC-2min  68ºC-1min | RSV: 737 AdV: 520 HMNV: 750 HBoV: 609 |
|  |  | RSVAB2 | AGGTGTWGTTACACCTGCATTRACACTRAATTC | F |  |  |
|  |  | ADHEX1F | CAACACCTAYGASTACATGAA | Hexon |  |  |
|  |  | ADHEX1R | KATGGGGTARAGCATGTT | Hexon |  |  |
|  |  | HMNVS | GAGTCCTACCTAGTAGACAC | M |  |  |
|  |  | HMNVA | TTGTYCCTTGRTGRCTCCA | M |  |  |
|  |  | HBOV1f | CACAGGAGCMGGAGYCGCAG | NP1-VP1/VP2 |  |  |
|  |  | HBOV1r | CCAAGATATYTRTATCCAGG | NP1-VP1/VP2 |  |  |
| Nested |  |  |  |  |  |  |
|  | Multiplex I^1^ Influenza A, B, C | FluAB3 | GATCAAGTGAKMGRRAGYMGRAAYCCAGG | NP | 95ºC-4 min 35 cycles:  94ºC-30sec  55ºC-1min  72ºC-30sec | Flu A: 301 FluB: 226 Flu C: 111 |
|  |  | FluC3 | AAATTGGAATTTGTTCCTTTCAAGGGACA | NP |  |  |
|  |  | FluAC4 | TCTTCAWATGCARSWSMAWKGCATGCCATC | NP |  |  |
|  |  | FluB4 | CTTAATATGGAAACAGGTGTTGCCATATT | NP |  |  |
|  |  |  |  |  |  |  |
|  | Multiplex II^2^ PIV, CoV, EV, RV | 3-PIV13 | ACGACAAYAGGAARTCATGYTCT | HA | 958C-4 min 35 cycles:  94ºC-30 sec  55ºC-1 min 72ºC-30 sec | PIV1: 439 PIV3: 390 PIV2: 297 PIV4AB: 174 HCoV 229E: 630 HCoV-OC43 587 EV: 226 RV: 110 |
|  |  | 4-PIV1 | GACAACAATCTTTGGCCTATCAGATA | HA |  |  |
|  |  | 4-PIV3 | GAGTTGACCATCCTYCTRTCTGAAAAC | HA |  |  |
|  |  | 3-PIV24 | CYMAYGGRTGYAYTMGAATWCCATCATT | HA |  |  |
|  |  | 4-PIV2 | GCTAGATCAGTTGTGGCATAATCT | HA |  |  |
|  |  | 4-PIV4 | TGACTATRCTCGACYTTRAAATAAGG | HA |  |  |
|  |  | 3-HcoV | TTGTGCGCAATGTTATAAWGGYAT | SP |  |  |
|  |  | 4-HcoV | GATAATRTGAGTRCCATTWCCACA | SP |  |  |
|  |  | 3-EV/RV | ACCRASTACTTTGGGTRWCCGTG | 50 |  |  |
|  |  | 4-EV/RV | CTGTGTTGAWACYTGAGCICCCA | 50 |  |  |
|  |  |  |  |  |  |  |
|  | Multiplex III^3^ RSV, AdV, HMPV^4^,HBoV^5^ | RSVA3 | TTATACACTCAACAATRCCAAAAAWACC | F | 95ºC-4 min 35 cycles:  94ºC-30 sec  55ºC-1 min  72ºC-30 sec | RSVA: 363 RSVB: 611 AdV: 475 HMNV: 718 HBoV: 243 |
|  |  | RSVA4 | AAATTCCCTGGTAATCTCTAGTAGTCTGT | F |  |  |
|  |  | RSVB3 | ATCTTCCTAACTCTTGCTRTTAATGCATTG | F |  |  |
|  |  | RSVB4 | GATGCGACAGCTCTGTTGATTTACTATG | F |  |  |
|  |  | ADHEX1R | CCCITTYAACCACCACCG | Hexon |  |  |
|  |  | ADHEX2R | ACATCCTTBCKGAAGTTCC | Hexon |  |  |
|  |  | HMNVS | GAGTCCTACCTAGTAGACAC | M |  |  |
|  |  | HMNVA2 | TCTTGCAKATYYTRCTKATGCT | M |  |  |
|  |  | HBOV2f | GTGGTGTGGGTTCTACTGGC | NP1-VP1/VP2 |  |  |
|  |  | HBOV2r | CTACGGTACACATCATCCCAG | NP1-VP1/VP2 |  |  |
|  |  |  |  |  |  |  |

^1^Coiras MT et al. , J Med Virol. 2003;69:132-44; ^2^Coiras MT et al. J Med Virol 2004;72:484-95; ^3^Calvo C et al. Acta Ped 2010;99:883-7; ^4^Lopez-Huertas et al. J Virol Methods 2010; 1-7; ^5^Pozo et al. J ClinVirol. 2007;40:224–228
